# Establishment of transient gene expression systems in protoplasts from *Liriodendron* hybrid mesophyll cells

**DOI:** 10.1371/journal.pone.0172475

**Published:** 2017-03-21

**Authors:** Ailing Huo, Zhenyu Chen, Pengkai Wang, Liming Yang, Guangping Wang, Dandan Wang, Suchan Liao, Tielong Cheng, Jinhui Chen, Jisen Shi

**Affiliations:** 1 Co-Innovation Center for the Sustainable Forestry in Southern China, Nanjing Forestry University, Nanjing, China; 2 School of Life Science, Huaiyin Normal University, Huaian, China; 3 Key Laboratory of Forest Genetics and Biotechnology, Ministry of Education, Nanjing Forestry University, Nanjing, China; 4 College of Biology and the Environment, Nanjing Forestry University, Nanjing, China; Wuhan University, CHINA

## Abstract

*Liriodendron* is a genus of the magnolia family comprised of two flowering tree species that produce hardwoods of great ecological and economic value. However, only a limited amount of genetic research has been performed on the *Liriodendron* genus partly because transient or stable transgenic trees have been difficult to produce. In general, transient expression systems are indispensable for rapid, high-throughput screening and systematic characterization of gene functions at a low cost; therefore, development of such a system for *Liriodendron* would provide a necessary step forward for research on Magnoliaceae and other woody trees. Herein, we describe an efficient and rapid protocol for preparing protoplasts from the leaf mesophyll tissue of a *Liriodendron* hybrid and an optimized system for polyethylene glycol–mediated transient transfection of the protoplasts. Because the leaves of the *Liriodendron* hybrid are waxy, we formulated an enzyme mix containing 1.5% (w/v) Cellulase R-10, 0.5% (w/v) Macerozyme R-10, and 0.1% (w/v) Pectolyase Y-23 to efficiently isolate protoplasts from the *Liriodendron* hybrid leaf mesophyll tissue in 3 h. We optimized *Liriodendron* protoplast transfection efficiency by including 20 μg plasmid DNA per 10^4^ protoplasts, a transformation time of 20 min, and inclusion of 20% (w/v) polyethylene glycol 4000. After integrating the *Liriodendron WOX1* gene into pJIT166-GFP to produce a *WOX1*-GFP fusion product and transfecting it into isolated protoplasts, Lh*WOX1*-GFP was found to localize to the nucleus according to its green fluorescence.

## Introduction

Plant transient-expression systems provide platforms for rapid, high-throughput screening and systematic characterization of gene functions. Importantly, when coupled with rapid “omics” technologies, such platforms have been used to study subcellular locations of various proteins [1, 2], promoter activities [3], protein-protein interactions [4, 5], signal transduction [6, 7], gene functions, and production of biologically derived compounds [8, 9]. Methods for transient gene expression have mainly involved *Agrobacterium tumefaciens*–mediated transformation [10], biolistic bombardment techniques [11], and polyethylene glycol (PEG)-mediated or electroporation-mediated protoplasts transfection [7]. Efficient PEG-mediated transient expression systems in protoplasts are commonly used and have been established in herbaceous model plants, e.g., *Arabidopsis thaliana* and *Nicotiana benthamiana*, with transfection efficiencies of 60–90% [12, 13]. A method for efficient transient transfection of Populus protoplasts has been established [2, 14, 15] and provides a model system for the study of woody plants in general.

Magnoliids are the third largest group within Mesangiospermae and are taxonomically defined as primitive angiosperms according to their morphological characteristics and conserved nuclear genes [16]. Although angiosperm phylogenetics remains ill defined, recent research has placed magnoliids at a primitive position on the angiosperm phylogenetic tree [17–19]. Therefore, deciphering the biological functions of genes in Magnoliaceae would significantly improve our knowledge of angiosperm phylogenetics in general. The genus *Liriodendron* in Magnoliaceae consists of two species, *L*. *tulipifera* and *L*. *chinense*, which are large deciduous trees. Both are common horticultural trees and provide high-quality timber. A *Liriodendron* hybrid was first produced by cross-fertilization of *L*. *chinense* and *L*. *tulipifera* by Ye and colleagues in 1963. The hybrid has superior physical characteristics, e.g., it grows more rapidly than do the parents, it readily adapts to changes in the environment, and has fewer naturally occurring pests than do its parents [20].

Notably, prior to this report, an efficient transient expression system using *Liriodendron* protoplasts did not exist, making it possible to characterize *Liriodendron* gene function only in heterologous systems, which can influence expression of the transferred genes. For example, Zhang and colleagues found that certain Arabidopsis markers were ambiguously localized or partial mislocalized in a heterologous rice system [21]. The proteins encoded in certain Arabidopsis genes induced in a tobacco system also mislocalized [22]. Therefore, establishment of a transient gene expression system using *Liriodendron* hybrid protoplasts would avoid the problems associated with *Liriodendron* gene expression in a heterologous system and, therefore, would avoid problems associated with such heterologous systems and, therefore, should yield a versatile tool with which to study *Liriodendron* gene functions at the cellular level and allow the introduction of potentially advantageous genes during *Liriodendron* breeding.

Because *Liriodendron* leaves are very waxy, it has been difficult to rapidly and directly isolate mesophyll protoplasts from them even though *Liriodendron* leaves derived from plantlets and grown in tissue culture have been an abundant and convenient source of protoplasts. Merkle and Sommer [23] have isolated protoplasts from cultures of suspended *L*. *tulipifera* cells, and Wang [24] isolated protoplasts from *L*. *tulipifera* cell suspensions and leaves; however, both procedures required a digestion time >10 h. Therefore, we developed and present herein a rapid protoplast-isolation procedure from *Liriodendron* hybrid leaf mesophyll tissue with optimized transfection parameters, which can serve as a model protoplast transient transfection system for Magnoliaceae.

Achieving rapid growth of plants as well as lateral organ differentiation are important issues needed for improving plant development because, in addition to being directly related to the effect on parts of a plant organ and morphogenesis, these considerations affect plant size and harvesting economics. Therefore, recent functional genomics research on plants has focused more attention on these concerns. Wushchel-like homeobox (*WOX*) genes are plant-specific homeodomain transcription factors and are needed for apical meristem development [25, 26]. To test our *Liriodendron* hybrid protoplast expression system, we first cloned *Liriodendron WOX1* (Lh*WOX1*) using the *Liriodendron* hybrid transcriptome sequence information, then integrated Lh*WOX1* into pJIT166-GFP (green fluorescent protein) to encode for the Lh*WOX1*-GFP fusion product, transformed the plasmid into the protoplasts, and characterized the subcellular location of the green fluorescence.

## Materials and methods

### Plant materials

Plantlets were collected from a *Liriodendron* hybrid somatic embryogenesis system that had been established in our lab [27]. Embryogenic callus induced by immature seeds obtained from the control-pollinated cores (hybrid between *L chinense* and *L*. *tulipifera*) were maintained on agar infused with inducing medium. Next, the embryogenic cells were cultured in suspension, and samples of these cells were germinated on the solid medium described above in a light-controlled growth room to produce plantlets.

### Construction of the GFP-fusion product vector

**Fig 1:**
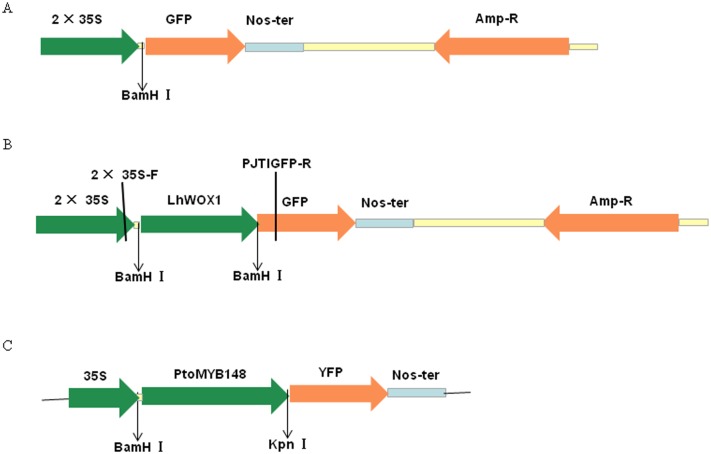
Vector schematics. (A) PJIT166-GFP, plasmid encoding GFP. (B) PJIT166-Lh*WOX1*-GFP, plasmid encoding Lh*WOX1*-GFP. (C) Pto*MYB*148-YFP, plasmid encoding Pto*MYB*148-YFP (*MYB*148 encodes a *Populus tomentosa* transcription factor). Abbreviations: 35S, the 35S promoter region of the cauliflower mosaic virus; 2 × 35S, tandem array of the 35S promoter region; NosT, the terminator region of the nopaline synthase gene; Amp-R, the ampicillin resistance gene; GFP, green fluorescent protein; YFP, yellow fluorescent protein; BamHI, KpnI, *BamH*I and *Kpn*I restriction sites, respectively; Lh*WOX1*, the *Liriodendron* hybrid transcription factor *WOX1* gene; Pto*MYB*148, the *Populus tomentosa MYB* transcription factor.

PJIT166-GFP is a high-copy vector driven by a double 35S cauliflower virus promoter. It harbors the GFP gene and a terminal nopaline synthase sequence. Ampicillin served as the bacterial selection marker. To test whether product of exogenously introduced genes would be targeted to their proper subcellular locations, we inserted the Lh*WOX1* open-reading frame upstream of GFP and used its expression as the test system. Transient expression vector of Yellow fluorescent protein (YFP)-tagged fusions of Pto*MYB148*-YFP (provided by Dr. Jianhua Wei, Beijing Agro-Biotechnology Research Center) was targeted to the nucleus when expressed in Arabidopsis leaf protoplasts, too was used to assess the ability of the expression system to target the product of an exogenously introduced gene to the appropriate location[28]. Primers used to prepare the Lh*WOX1*-containing plasmid were 5’-GGATCCATGTGGATGATGGGTTGTAGAGATG-3’ (forward direction) and 5’-GGATCCATTCTTCAATGGGAGAAACTGGATG-3’ (reverse direction). PCR was performed using Taq DNA polymerase (TaKaRa). Lh*WOX1* was inserted upstream of GFP in pJIT166-GFP using T4 DNA ligase (NEB) after digestion of the vector with *Bam*HI (NEB; restriction sites are underlined in the primer sequences). The purified plasmid was sequenced with the 2 × 35S-F and PJTIGFP-R primers provided by Thermo Fisher Scientific, which confirmed successful Lh*WOX1* fusion (Fig 1). PJIT166-Lh*WOX1-GFP* was propagated in *Escherichia coli* DH-10B, with the transfection-ready plasmid DNA first prepared using Qiagen Plasmid Midi Prep kit reagents. The concentration and quality of the DNA used for transfection were determined using a Nanodrop 2000 UV-Vis Spectrophotometer (Thermo Scientific). The plasmid concentration was adjusted to 2 μg/μL and stored at –20°C until used.

### Protoplast isolations

Plantlets were grown on three-fourths concentrated Murashige and Skoog medium under a 16-h florescent light/8-h dark cycle at 23°C. After picking healthy, well-expanded leaves from plantlets (20 d of age), the leaves were cut into 1–2 mm strips. 100 mg of the cut leaves were immediately and gently transferred into a solution (10 mL) containing 0.5 M mannitol, 20 mM MES, pH 5.7, 20 mM KCl, 0.1% (w/v) bovine serum albumin, 10 mM CaCl_2_, and one of the enzyme cocktails described in Table 1. The digests were incubated in the dark at 50 rpm, 28°C for 3 h. After digestion, each individual solution was diluted with 10 mL of 2 mM MES, pH 5.7, 154 mM NaCl, 125 mM CaCl_2_, and 5 mM KCl to quench the digestion. Each solution was individually filtered through a 50-μm sifter and washed with 2 mL of the quench solution. The filtrates were centrifuged at 200g in round-bottomed tubes for 2 min to pellet the protoplasts. After removing most of each supernatant by pipetting, the protoplasts were suspended in 500 μL of the quench solution by gentle swirling and counted under a microscope using a hemocytometer. The remaining protoplasts were kept on ice for 30 min. To optimize the number of protoplast isolated, the digestion time (1, 2, 3, 4, 5, and 6 h) and the mannitol concentration (0.3 M–0.8 M) were assessed. The efficiency of protoplast isolation was calculated as protoplast yield and viability. The protoplast yield was defined as the total number of protoplasts per fresh leaf mass. The cells were counted at least three times for each sample. Protoplast viability was determined by counting the number of protoplasts stained with fluorescein diacetate (Sigma) under a fluorescence microscope(Imager D2, Zeiss) and is expressed as the percentage of fluorescing protoplasts to the total number of protoplasts[29].

**Table 1:** Effects of different enzyme combinations on protoplast isolation.

Trial number	Enzyme and concentration ^a^						Yield (per g FW)^b^	Viability (%)
	Cellulase R-10 (%)	Macerozyme R-10 (%)	Pectolyase Y-23 (%)	Hemicellulase (%)	Pectinase P0024 (%)	Pectinase T2445 (%)		
1	1.5	0.5					2 × 10^4^ ± 5 × 10^3^	97 ± 3
2	3	1					4 × 10^4^ ± 3 × 10^3^	91 ± 3
3	1.5	0.5	0.25				8 × 10^6^ ± 3 × 10^5^	89 ± 4
4	1.5	0.5			0.25		2.5 × 10^4^ ± 4 × 10^3^	89 ± 3
5	1.5	0.5				0.25	2.3 × 10^4^ ± 3 × 10^3^	87 ± 5
6	1.5	0.5	0.1				1.2 × 10^7^ ± 3 × 10^6^	95 ± 4
7	2		0.2	0.1			4 × 10^6^ ± 6 × 10^5^	99 ± 1

^a^ Cellulase R-10, Macerozyme R-10, and Pectolyase Y-23 were purchased from Yakult Honsha Co., Ltd.; Hemicellulase were purchased from Sigma; Pectinase P0024 and Pectinase T2445 were purchased from Nanjing Bo-wide Technology Co., Ltd.

^b^g FW, gram fresh weight.

### Protoplast transfections

PEG-mediated transfections were performed with modifications [12]. Before initiating each transfection, protoplasts were collected after removing most of the quench solution by pipetting without touching the protoplast pellet. Protoplasts were then suspended in 4 mM MES, pH 5.7, 0.5 M mannitol, and 15 mM MgCl_2_ at 10^4^, 10^5^, 10^6^, or 10^7^ per mL at room temperature. For each transfection assay, 100 μL of each protoplast suspension (containing 10^3^, 10^4^, 10^5^, or 10^6^ protoplasts) was added into 10 μL of water containing 20 μg plasmid in a 2-mL microfuge tube and mixed gently. Next, an equal volume (110 μL) of a freshly prepared solution containing 0.2 M mannitol, 100 mM CaCl_2_, and PEG 4000 (30–50%, w/v) was immediately added to a plasmid/protoplast solution and mixed by gentle inversion. To optimize the transfection duration, mixtures were incubated at room temperature for 10, 20, 30, or 40 min, after which 440 μL of the quenching solution was slowly added. The protoplasts were collected by centrifugation at 200g for 2 min to remove the supernatant, suspended in 1 mL of 4 mM MES, pH 5.7, 0.5 M mannitol, 20 mM KCl, and then pipetted into the wells of six-well culture plates. Next, the protoplasts were incubated in the dark for 18–48 h at 23°C. After incubation, protoplasts were concentrated by centrifugation at 200g for 2 min to remove 800 μL supernatant and re-suspend protoplasts with the remaining solution. Protoplasts were observed under a ZEISS Imager D2 fluorescence microscope (Carl Zeiss, Germany). Transfection efficiency was calculated as the percentage of fluorescing protoplasts to total protoplasts. In addition, PJIT166-Lh*WOX1*-GFP was bombarded into single-layer onion epidermal cells using the Biolistic PDS-1000/He Particle Delivery system (Bio-Rad) according to the manufacturer’s instructions, and the location of the fluorescence recorded in the same manner.

### Statistical analysis

All experiments were independently performed three times with three replicates per experiment. Duncan’s multiple range test in SPSS software Version 19 was used to analyze the difference between the samples. A p-value of <0.05 was considered significant.

## Results and discussion

### Isolation of *Liriodendron* hybrid protoplasts from leaf mesophyll cells

**Fig 2:**
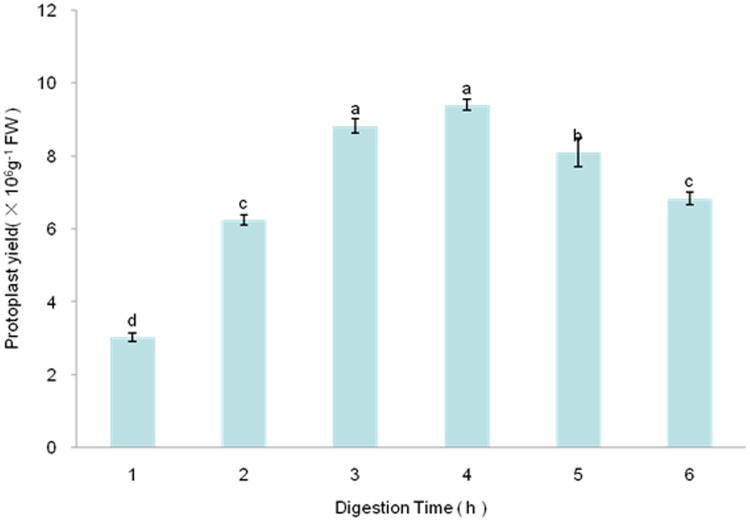
Protoplast isolation vs. digestion time. Incubation mixture contained 1.5% (w/v) Cellulase R-10, 0.5% (w/v) Macerozyme R-10, 0.1% (w/v) Pectolyase Y-23, and 0.4 M mannitol.

**Fig 3:**
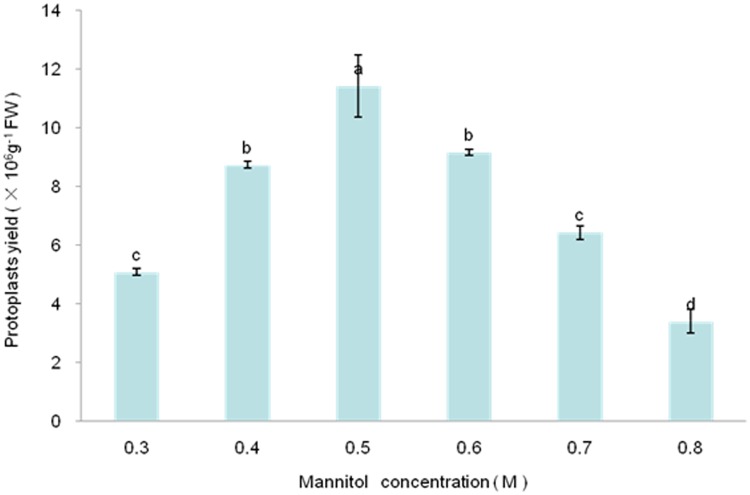
Influence of mannitol concentration on the efficiency of isolating *Liriodendron* hybrid leaf protoplasts. Incubation mixtures contained 1.5% (w/v) Cellulase R-10, 0.5% (w/v) Macerozyme R-10, 0.1% (w/v) Pectolyase Y-23 and one of various concentrations of mannitol. Error bars represent standard errors (n = 3). Different letters above bars indicate a statistical difference at p≦0.05 among samples according to Duncan’s multiple range tests.

Plants protoplasts are important and effective biochemical tools for plant gene expression assays [30]. From the time Cocking [31] first used an enzyme (cellulase) to isolate protoplasts from the root tips of tomato seedlings and ever since protoplasts have been isolated from many types of plants and their tissues, a variety of enzymes have been used for protoplast isolation [32]. Cellulase R-10 and Macerozyme R-10 in combination have been commonly used. However, differences in the compositions of cell walls from different source materials require the optimization of each digestion. For example, because extensive intercellular spaces are found in *Bienertia sinuspersici* leaves, their loosely packed chlorenchyma cells can be readily released when leaves are gently squeezed in a mortar and pestle [33]. The use of only Cellulase R-10 efficiently isolates *B*. *sinuspersici* mesophyll protoplasts. Homogeneous layers of epicuticular wax are found in *Liriodendron*, *Ginkgo biloba*, and *Magnolia grandiflora* leaves [34], which makes it difficult to physically remove the leaf epidermal layer as has been done for Arabidopsis, tobacco, cereal, and poinsettia leaves [35, 36]. We formulated seven enzyme cocktails to improve isolation of protoplasts from *Liriodendron* hybrid leaves (Table 1). However, the commonly used combination of Cellulase R-10 and Macerozyme R-10 cannot efficiently and rapidly isolate mesophyll protoplasts from the *Liriodendron* hybrid leaf (Table 1, runs 1 and 2). Fortunately, we found that Pectolyase Y-23 is essential for isolating protoplasts from *Liriodendron* hybrid leaf (Table 1, runs 3, 6, and 7). By including Pectolyase Y-23, the protoplast yield was significantly greater than when the enzyme was absent, which implies that Pectolyase Y-23 may be crucial for digestion of the cell wall of wax-rich leaves. Tan and colleagues used a combination of Cellulase C2605 and Pectinase P2601 (both from Sigma) to release protoplasts from Populus mesophyll cells [14]. Because enzyme concentrations influence protoplast yield and viability, we optimized the Pectolyase Y-23 concentration and found that 0.1% (w/v) Pectolyase Y-23 was optimum, whereas a greater concentration of Pectolyase Y-23 (0.25% w/v) did not improve the yield. The enzyme digestion period used to isolate protoplasts from different plant tissues has varied considerably (S1 Table). Wu and colleagues incubated *A*. *thaliana* mesophyll cells for 20 to 60 min [35], whereas Pitzschke and colleagues used an incubation time of 16 h to isolate protoplasts from *Euphorbia pulcherrima* red leaves [36]. Our work revealed that the protoplast yield increased with digestion time from 1 to 4 h, but then the yield and viability decreased accompanied by an increased amount of cell debris (Fig 2). Because the protoplast yield did not differ significantly for the 3 h and 4 h time points, we used an incubation time of 3 h for protoplast isolation. An osmoticum is required when preparing protoplasts to allow for cell plasmolysis, which facilitates wall digestion, and for the resulting protoplasts to counteract turgor pressure. Osmoticums include the sugars d-mannitol, d-glucose, sucrose, and d-sorbitol. Mannitol has been commonly used for protoplast isolation in the concentration range 0.4–0.6 M (S1 Table). We found that the use of 0.5 M mannitol in the digest resulted in a large yield of protoplasts (1.2 × 10^7^ g^–1^ FW) (Fig 3).

**Fig 4:**
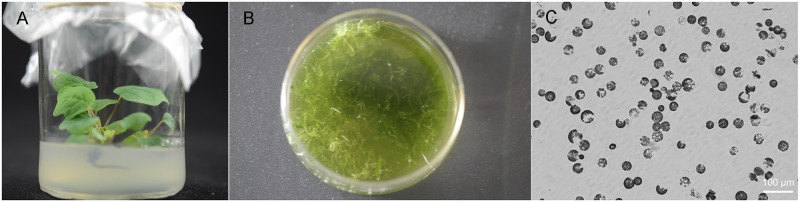
Isolated protoplasts from leaf mesophyll tissue of the *Liriodendron* hybrid. (A) Test tube–grown plantlets. (B) Cut leaves incubated with different enzyme mixtures. (C) Protoplasts from leaf mesophyll tissue, Scale bar = 100 μm, Images of the protoplasts were captured using an Imager D2 fluorescence microscope (Zeiss).

**Fig 5:**
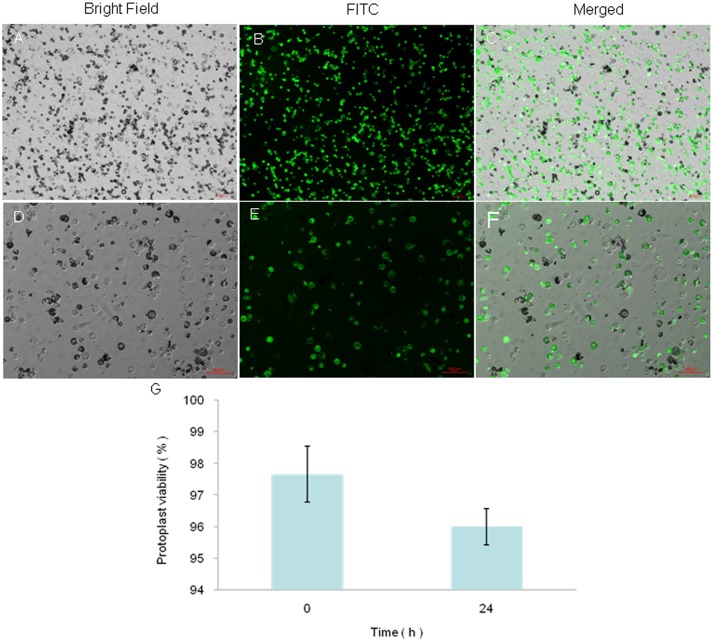
Fluorescein diacetate staining of protoplasts from leaf mesophyll tissue of the *Liriodendron* hybrid. (A–C) Protoplasts from mesophyll tissue. (D–F) Protoplasts after incubation for 24 h in the dark. (G) Protoplast viability after different incubation times defined as the ratio of the number of stained protoplasts to total protoplasts. Values are the mean ± standard error (bar) of three replicates.

Our optimized digestion condition included 0.5 M mannitol, 10 mM MES, pH 5.7, 20 mM KCl, 0.1% (w/v) bovine serum albumin, 10 mM CaCl_2_, 1.5% (w/v) Cellulase R-10, 0.5% (w/v) Macerozyme R-10, 0.1% Pectolyase Y-23, room atmospheric pressure, 28°C, 50 rpm, and an incubation period of 3 h, which gave a protoplast yield of ~1.2 × 10^7^/g FW. Their sizes were 10 μm to 50 μm in diameter, with the majority being between 15 μm and 30 μm (Fig 4). Their number was sufficient for >200 transfection experiments (10^4^ protoplasts per transfection). The viability of the protoplasts was 97% as judged by fluorescein diacetate staining. Viability remained excellent even after protoplasts had been incubated in the dark for >24 h (Fig 5). Given the aforementioned characteristics, our protoplast preparation provided sufficient experimental material and viability for carrying out protoplast culture and transfections.

### Optimization of *Liriodendron* hybrid mesophyll protoplast transfection

PEG-mediated transient transformation systems involving protoplasts from many different plant species have been established and include those of poplar [2, 14], cotton [37], poinsettia [36], rice [21], wheat [38], and barley [39]. Characterization of these established protoplast transient expression systems indicated that the concentration and purity of the transfecting plasmid, plasmid-to-protoplast ratio, PEG concentration, and transfection duration influence transfection efficiency (S2 Table).

The protoplast to plasmid DNA ratio is an important factor affecting transformation efficiency. Previous studies found that the optimal ratio ranged from 1 × 10^4^ to 7 × 10^5^ protoplasts per 10 μg plasmid DNA, indicating significant differences when using different protoplast transformation systems. Pitzschke and colleagues used 0.7 μg plasmid DNA per 25,000 to 50,000 protoplasts and observed a >70% transfection efficiency [36]. Yoo and colleagues used 10 to 20 μg of plasmid DNA per 2 × 10^4^ Arabidopsis protoplasts and achieved a transfection efficiency >90% [12]. In general, the amount of plasmid DNA used has been between 10 and 20 μg in mesophyll protoplast transient expression systems (S2 Table). To determine the optimal transfection conditions for the *Liriodendron* hybrid mesophyll protoplasts, we transfected the protoplasts with the GFP-encoding vector PJIT166-GFP and studied the effects of protoplast number, PEG 4000 concentration, and transfection time on transfection efficiency. The experiments were carried out in the sequence presented below. After each optimization step, the optimized condition was incorporated into the next set of optimization experiments.

**Fig 6:**
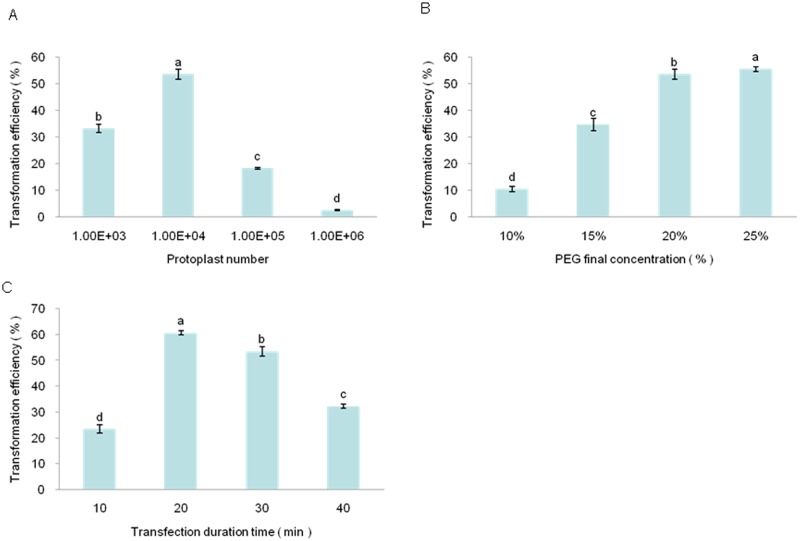
Optimization of *Liriodendron* hybrid protoplast transfection. (A) Effect of protoplast number on transfection efficiency. (B) Effect of PEG 4000 concentration on transfection efficiency. (C) Effect of transfection time on transfection efficiency. Error bars represent standard errors (n = 3). Different letters above bars indicate a statistical difference at p ≤ 0.05 among samples according to Duncan’s multiple range test.

The first optimized variable was the number of protoplasts. The amount of plasmid was held constant at 20 μg, and the number of protoplasts was varied between 10^3^ and 10^6^. At 10^3^ protoplasts, the transfection percentage was 33.29%. When the protoplast number was 10^4^, the transfection percentage was 53.55%. At 10^6^ protoplasts, however, the transfection percentage decreased to 2.67% (Fig 6A). Therefore, in the presence of 20 μg plasmid DNA, 10^4^ protoplasts provided the greatest transfection efficiency, and this quantity was used in subsequent experiments.

PEG is widely used to induce protoplast transfection, as it interacts with protoplasts and allows the target DNA to enter [40]. Consequently, the molecular weight of different PEG preparations and the PEG concentration influence transfection efficiency. PEG 4000, PEG 6000, and PEG 8000 are the most commonly used forms of PEG for transfection of protoplasts from many plant species, with PEG 4000 being most widely used. Some previous studies suggested that up to a maximum concentration, there was a linear relationship between the PEG concentration and the number of transfected protoplasts[15, 41]. The second variable optimized was the PEG 4000 concentration, with concentrations of 10%, 15%, 20%, and 25% (w/v) tested. As the PEG 4000 percentage increased, the transfection efficiency also increased (Fig 6B). At 25% (w/v) PEG 4000, the efficiency was the greatest (~55.49%), but many protoplast fragments were seen under the microscope. No significant difference in transfection efficiency was found when 20% (w/v) or 25% (w/v) PEG 4000 was used. Therefore, we used a PEG 4000 concentration of 20% (w/v) as the optimum concentration.

The transfection time also influences transfection efficiency of protoplasts from many plant species. The effect of time on transfection efficiency was assessed for 10, 20, 30, and 40 min. At 10 min, the transfection efficiency was 23.53% and increased to a maximum value (60.65%) at 20 min. Protoplast fragments were seen at times >20 min accompanied by decreased transfection efficiency (Fig 6C). and perhaps, with time, the presence of PEG caused degradation of the protoplasts. Transient expression of pUC19 transient expression plamids in *Populus euphratica* Oliv. protoplasts was decreased by 40 min, with 20% PEG 4000 having caused a rapid decrease in protoplast viability [15]. Therefore, a transfection time of 20 min was employed in the optimized procedure.

### Subcellular location of transfected Lh*WOX1*-GFP

**Fig 7:**
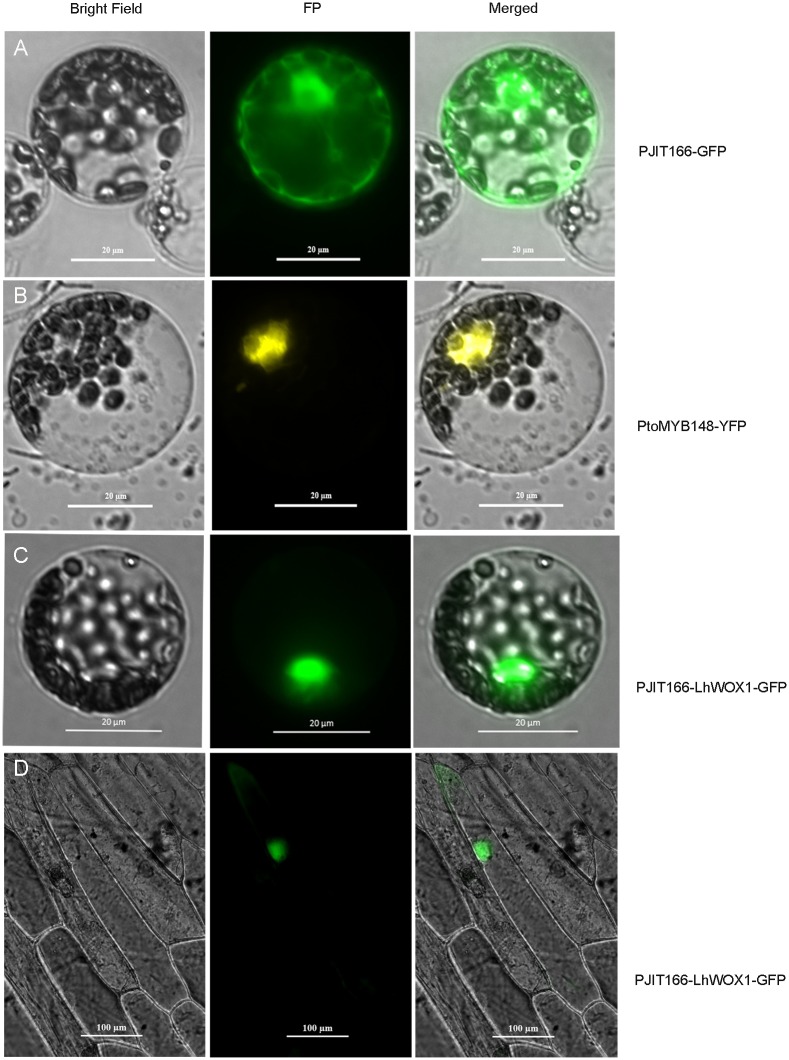
Subcellular localization of *LhWOX1*-*GFP*-induced fluorescence in protoplasts from *Liriodendron* hybrid leaves. (A) Transfection with PJIT166-GFP (the GFP-encoding parent vector) led to GFP fluorescence throughout the protoplast interior. (B) Transfection with Pto*MYB*148-YFP (the plasmid encoding Pto*MYB*148-YFP) led to YFP fluorescence the protoplast nuclei. (C) Transfection with PJIT166-Lh*WOX1*-GFP (the plasmid encoding Lh*WOX1*-GFP) led to GFP fluorescence in the protoplast nuclei. (D) Bombardment of Lh*WOX1*-GFP into onion epidermal cells led to GFP fluorescence in nuclei. (A–D) Panels, left to right: bright-field channel, fluorescence channel, and overlay.

To test whether the products of exogenously introduced genes would be targeted to their proper subcellular locations in the *Liriodendron* hybrid protoplasts, we individually transfected samples of the protoplasts using the optimized protocol with PJIT166-GFP, PJIT166-Lh*WOX1*-GFP, or Pto*MYB148*-YFP. GFP fluorescence associated with PJIT166-GTP was homogeneously distributed throughout the protoplasts (Fig 7A), whereas YFP fluorescence associated with Pto*MYB148*-YFP transfection was seen in the nucleus (Fig 7B), as expected given the location of *MYB*148 in *Arabidopsis* leaf protoplasts [27] and the fact that GFP fluorescence associated with PJIT166-Lh*WOX1*-GFP transfection was also seen in the nuclei of the protoplasts (Fig 7C). The nuclei of the onion epidermal cells also fluoresced green after being bombarded with Lh*WOX1*-GFP (Fig 7D). Because Lh*WOX1* was found in the nuclei, it might function as a transcription factor.

## Conclusions

We have established a convenient, fast and reliable transient gene expression system for *Liriodendron* hybrid protoplasts from mesophyll tissue. This system includes isolation with high yield and high viability from *Liriodendron* hybrid leaves, PEG-mediated protoplast transfection, and fluorescent protein detection of the transfected protoplasts. The isolation and transfection process took 4 h, and results could be obtained within 24 h. Transfected of known markers proved the reliability of this transient gene expression system. This is the first transient gene expression system for *Liriodendron* protoplasts. With knowledge of the *Liriodendron* genome and transcriptome sequences, and in combination with other available technologies including siRNA, miRNA, and CRISPR, it should play an important role in the elucidation of gene functions and metabolic pathways for trees.

## Supporting information

S1 Table Optimized conditions for protoplast isolation from different plant organs. (DOCX)

S2 Table Summary of optimal parameters for transient transfection of different plant protoplasts. (DOCX)

S1 References (DOCX)
