# Supplementary material for: Establishment of transient gene expression systems in protoplasts from Liriodendron hybrid mesophyll cells
Source: PLoS One. 2017 Mar 21;12(3):e0172475. doi: 10.1371/journal.pone.0172475 (PMC5360215; doi:10.1371/journal.pone.0172475)
Supplement: S1 Table — (DOCX) [file pone.0172475.s001.docx]

| **S1 Table. Optimized conditions for protoplast isolation from different plant organs.** |
| --- |
| \| Plant, Tissue \| Enzymes \| Incubation period \| Osmoticum \| Yield/(g FW) \| Method \| References \| \| --- \| --- \| --- \| --- \| --- \| --- \| --- \| \| *Arabidopsis* *thaliana*, leaf \| 1.5% Cellulase R-10, 0.4% Macerozyme R-10 \| 4–5 h \| 0.4 M mannitol \| 10 ^7^ \| Cutting \| [12] \| \| *Arabidopsis thaliana*, leaf \| 1% CellulaseR-10,  0.25% Macerozyme R-10 \| 20–60 min \| 0.4 M mannitol \| 3.0 ± 0.3 × 10^7^ \| Tape \| [35] \| \| hybrid poplar, leaf \| 2% Cellulase C2605,  0.5% Pectinase P2611 \| 3 h \| 0.6 M mannitol \| 1 × 10^8^ \| Cutting \| [14] \| \| *Populus tremula*, leaf \| 3% CellulaseR-10,  0.8% Macerozyme R-10 \| 5 h \| 0.4 M mannitol \| 1 × 10^7^ \| Cutting \| [2] \| \| *Brachypodium distachyon*, leaf \| 1.5% CellulaseR-10, 0.75% Macerozyme R-10 \| 3 h \| 0.6 M mannitol \| 1.7 × 10 ^7^ \| Cutting \| [42] \| \| *Euphorbia pulccherrima*, leaf \| 1% CellulaseR-10,  0.25% Macerozyme R-10 \| 16 h \| 0.4 M mannitol \| 1–1.5 × 10^8^ \| Tape \| [36] \| \| *Oryza sativa*, green tissue \| 1.5% Cellulase RS,  0.75% Macerozyme R-10 \| 4–5 h \| 0.6 M mannitol \| 1 × 10 ^7^ \| Cutting \| [21] \| \| *Panicum virgatum*, leaf \| Food-grade Rohament CL 1320 ECU, Rohapect 10L 840 ADJU, and Rohapect UF 0.0065 ADJU \| 3 h \| 0.6 M mannitol \| 1.6×10 ^6^ \| Cutting \| [43] \| \| *Bienertia sinuspersici*, leaf \| 1.5% cellulase R-10 \| 4 h \| 0.7 M sucrose \| 1.8 ± 0.29× 10 ^5^ \| Cutting \| [33] \| \| *Zea mays*, nucellus \| 1.5% Cellulase RS,  0.75%Macerozyme R-10 \| 4–5 h \| 0.6 M mannitol \| - \| Cutting \| [44] \| \| *Populus euphratica,* suspension cells \| 4% cellulaseR-10,  0.5% pectinase, 0.2% hemicellulase \| 4 h \| 0.5 M mannitol \| 8×10 ^7^ \| - \| [15] \| \| *Vitis vinifera*, suspension cells \| 2% CellulaseR-10,  1% Macerozyme R10 \| 4 h \| 0.5 M mannitol \| 3×10 ^7^ ± 1×10 ^7^ \| - \| [41] \| \| *Liriodendron* Hybrids, leaf \| 1.5% Cellulase R-10, 0.5% Macerozyme R-10, 0.1% Pectolyase Y-23 \| 3 h \| 0.5 M mannitol \| 1.2×10 ^7^ \| Cutting \| This study \| |
