# Supplementary material for: Establishment of transient gene expression systems in protoplasts from Liriodendron hybrid mesophyll cells
Source: PLoS One. 2017 Mar 21;12(3):e0172475. doi: 10.1371/journal.pone.0172475 (PMC5360215; doi:10.1371/journal.pone.0172475)
Supplement: S2 Table — (DOCX) [file pone.0172475.s002.docx]

| **S2 Table.** **Summary of optimal parameters for transient transfection of different plant protoplasts.** |
| --- |
| \| Plant \| DNA  amount \| Protoplast number \| PEG molecular weight  and concentration \| Transfection duration time (min) \| Efficiency \| References \| \| --- \| --- \| --- \| --- \| --- \| --- \| --- \| \| *Arabidopsis thaliana* \| 10–20 μg \| 2 ×10^4^ \| PEG 4000, 20% \| 15 \| 60–90% \| [12] \| \| *Arabidopsis thaliana* \| 30 μg \| 5 × 10^4^ \| PEG 4000, 20% \| 5 \| 60–66% \| [35] \| \| hybrid poplar \| 10 μg \| 8 × 10^4^ \| PEG 4000, 15% \| 15 \| >80% \| [14] \| \| *Populus tremula* \| 20 μg \| 1 × 10^5^ \| PEG 4000, 20% \| 5 \| >70% \| [2] \| \| *Brachypodium distachyon* \| 20 μg \| 1 × 10^5^ \| PEG 4000, 20% \| 5-10 \| >60% \| [42] \| \| *Euphorbia pulccherrima* \| 0.7 μg \| 2.5–5 × 10^4^ \| PEG 4000, 20% \| 10 \| >70% \| [36] \| \| *Oryza sativa* \| 5 μg \| 2 × 10^5^ \| PEG 4000, 20% \| 10–20 \| 53–75% \| [21] \| \| *Panicum virgatum* \| 10 μg \| 2 × 10^5^ \| PEG 4000, 25% \| 10 \| 30.4% \| [43] \| \| *Bienertia sinuspersici* \| 5 μg \| 1.5× 10^4^ \| PEG 4000, 20% \| 5 \| 80% \| [33] \| \| *Zea mays* \| 5–10 μg \| 2 × 10^5^ \| PEG 4000, 20% \| 15 \| - \| [44] \| \| *Populus euphratica* \| 20 μg \| 1 × 10^5^ \| PEG 4000, 20% \| 20 \| >50% \| [15] \| \| *Vitis vinifera* \| 20 μg \| 4 × 10^5^ \| PEG 4000, 25% \| 30 \| 60% \| [41] \| \| *Liriodendron* Hybrids \| 20 μg \| 1× 10^4^ \| PEG 4000, 20% \| 20 \| 60% \| This study \| |
