# Supplementary material for: Establishment of transient gene expression systems in protoplasts from Liriodendron hybrid mesophyll cells
Source: PLoS One. 2017 Mar 21;12(3):e0172475. doi: 10.1371/journal.pone.0172475 (PMC5360215; doi:10.1371/journal.pone.0172475)
Supplement: S1 References — (DOCX) [file pone.0172475.s003.docx]

S1 References:

42. Hong S-Y, Seo PJ, Cho S-H, Park C-M. Preparation of leaf mesophyll protoplasts for transient gene expression in Brachypodium distachyon. Journal of Plant Biology. 2012;55(5):390-7.

43. Burris KP, Dlugosz EM, Collins AG, Stewart Jr CN, Lenaghan SC. Development of a rapid, low-cost protoplast transfection system for switchgrass (Panicum virgatum L.). Plant Cell Reports. 2015:1-12.

44. Chen J, Yi Q, Song Q, Gu Y, Zhang J, Hu Y, et al. A highly efficient maize nucellus protoplast system for transient gene expression and studying programmed cell death-related processes. Plant Cell Reports. 2015;34(7):1239-51.
